# The influence of hospital services on patient satisfaction in OPDs: evidence from the transition to a digital system in South Punjab, Pakistan

**DOI:** 10.1186/s12961-024-01178-8

**Published:** 2024-08-05

**Authors:** Shahida Kanwel, Zhiqiang Ma, Mingxing Li, Abid Hussain, Naila Erum, Saif Ahmad

**Affiliations:** 1 School of Management, Jiangsu University, Zhenjiang, 212013 People’s Republic of China; 2 Accounting Research Institute (HiCOE), Universiti Teknologi MARA (UiTM), Shah Alam, Malaysia; 3 Department of Public Administration, The Islamia University, Bahawalpur, Pakistan

**Keywords:** Pharmacy, Doctors, Nurses, OPDs, Public hospitals, Punjab, Pakistan

## Abstract

**Background:**

Pakistani’s health services delivery system has been rarely evaluated regarding patient satisfaction. This study examined the performance of the Pakistani health system from the perspective of doctor services (DS), digital payment system (DPS), nurses’ services (NS), laboratory services (LS), pharmacy services (PHS), registration services (RS), physical services (environmentally and tangible) and doctor-patient communication (DPC) about patient satisfaction. A random sampling technique was adopted for data collection.

**Methodology:**

The Social Science Statistical Package (SPSS), analysis of moment structures (AMOS), and structural equation modeling were used to analyze the data for reliability, validity, correlations, and descriptive findings. The 879 responses were used for study analysis.

**Results:**

The study revealed that patient satisfaction was found to be significantly affected positively by LS, PHS, DS, NS, and DPS, while DPC, RS, and PF were impacted non-significantly. Consequently, there is a considerable communication gap in the doctor-patient interaction, and Pakistan's healthcare system is confronted with a shortage of physical infrastructure and challenges in the digital system.

**Conclusion:**

Furthermore, the insufficient emphasis on registration services necessitates immediate action to improve the entire patient experience and satisfaction. Identifying these shortcomings has the potential to result in a healthcare system that is more efficient and focused on the needs of the patients.

**Supplementary Information:**

The online version contains supplementary material available at 10.1186/s12961-024-01178-8.

## Introduction

The World Health Organization issued a pandemic notice more than ten years before 2020, about the global spread of the H1N1 virus in 2009 [1]. The World Health Organization issued a fresh statement on March 11 warning about the COVID-19 pandemic as a result of the degree of contamination caused by a new coronavirus type with high infectious potential in 2020 [2]. Recently, the quality of healthcare has drawn too much attention after the crises of COVID-19 around the whole globe and coronavirus was a big factor in the shift towards a digital system regarding the services. The World Health Organization emphasized to all member countries for better delivery regarding the health sector [3]. The evaluation of hospital service delivery is closely related to both the performance of the health service providers and the effectiveness of the system. Medical costs, doctor-patient communication, emergency department, and laboratory services, are some factors measured by scholars for assessing patient satisfaction as well as the performance of the institution [4, 5]. The hospital personnel can provide better care and valves to patients for developing a strong healthcare delivery system [6, 7]. Previously defined by [8] that upgraded support, to achieve the health-related Millennium Development Goals, healthcare delivery must be prioritized. Since healthcare procedures are always evolving and enhancing, it is important to have a method of assessing results while also tracking patients' levels of contentment [9].

The Global Strategy on Digital Health (2020–2025) by the World Health Organization advocates that hospitals should improve their services with the national digital health architecture. Currently, the services of hospitals shifting to the channel of digital such as appointments of patients, doctor-patient communication, instructions from the pharmacy, and the payments of hospital or medicine bills. A digital hospital utilizes an electronic medical record (EMR) to accomplish its healthcare delivery objectives [10] and is increasingly becoming the standard approach to providing services in the OPDs for patient satisfaction [11]. The digital systems in healthcare settings have strongly transformed several aspects of services, ranging from administrative procedures to centralized patient care, which has impacted overall patient perceptions and satisfaction levels [12]. The term "patient satisfaction" refers to how a patient feels about various facets of their care [2]. The hospital's regular treatment and quality could be enhanced by utilizing information obtained from patient assessments. Academics may recognize it as a distinct factor for examining the quality of care delivered, particularly about the improvement of the institution [13]. The hospital’s regular treatment and quality could be enhanced by utilizing information obtained from patient assessments. Despite numerous research emphasizing the importance of identifying the specific measurements that influence patient satisfaction, there is currently no consensus on the most effective method for forecasting patient satisfaction [1, 14].

Service delivery is an essential aspect in determining the public health status, along with other components that form the social dimensions of health. Although the characteristics of an institution as well as the form of health services might change between countries, the following factors should be present in every well-structured health system to define the network of health service delivery and provision. The key factors to consider in healthcare are affordability, availability, comprehensiveness, effectiveness, and continuity of service. It means a systematic approach for health service organizations, where each level is considered through the lens of a local medical care system, which acts as a driver or motivator for the overall healthcare delivery system [15–17]. The qualities of service delivery are a major force for sustainability, policy-making, satisfaction, and business growth in the health sector (2020), because satisfaction leads to loyalty which is a positive sign. Based on research in the field of healthcare, multiple institutions provide services that are similar in structure but different in terms of quality. The healthcare system's overall success in institutions is significantly influenced by the exceptional quality of services offered by both medical and non-medical staff with the assistance of digital transitions [18]. The concept of satisfaction has changed the health sector which emphasizes service providers for better performance. Scholars measured patient satisfaction through convenience, information received and performance; distance from the hospital influenced patient satisfaction [19–22].

The document titled "Pakistan 2025 One Nation—One Vision" was developed by the Planning Commission of the Ministry of Planning, Development and Reform in 2014. The document identifies the key priorities for the nation's digitalization objectives. For example, it proposes that by 2025, all schools, colleges, and institutions should be digitized and delivered with digital systems. Furthermore, it places great importance on the implementation of digital transactions in several sectors, including education, health, business and commerce, agriculture, energy, and other public service areas, to achieve extensive digital connectivity [23]. There is no national legislation that specifically addresses health information, including health records and disease reporting. Developing expertise and digital tools within a healthcare system is essential for the efficient and effective provision of high-quality care. Integrating hospital information from a wide network and bridging the difference between knowledge and appropriate treatments, has the potential to transform healthcare delivery. Saudi Arabia’s Vision 2030 prioritizes enhancing the efficiency and effectiveness of the healthcare sector by employing information technology and digital transformation. Tanzania's Ministry of Health is working on providing health services to people through a digital system in the hospital [24].

Major barriers identified regarding the services of hospitals include the lack of capital, maintenance-related costs, attitudes of healthcare professionals, and unavailability of staff [20]. Besides this, technological, environmental, organizational, and human factors were also identified as the key issues for care provision of healthcare about patient satisfaction [21, 22]. The main challenges to effective healthcare delivery in Indonesia were identified as personnel attitude, insufficient communication training and skills, and limited administrative assistance for patients [25]. The key reason that led to decreased acceptability levels of health information technologies in Saudi Arabia was the absence of advanced technology for satisfaction [3]. Many developing nations are facing challenges in delivering sufficient healthcare services. Furthermore, Pakistan is confronted also significant challenges, such as excessive healthcare expenses, limited healthcare accessibility, inadequate hospital services, increasing outpatient department waiting times, substandard physical facilities, fragmented health information systems [25], and human resource shortage [26].

Poor healthcare delivery procedures lead to dissatisfied patients, which in turn leads to tragic events, financial losses, wasted time, compromised ethics, decreasing confidence in institutions, and increased public and community engagement in healthcare [27]. Pakistan, the world's sixth-most populous nation, has introduced several new healthcare delivery reforms. Prior studies have shown that Pakistan's healthcare delivery system is thriving, but issues like poor accessibility, convenience, physical environment issues, medical expenses, quality of care, long distances, lack of necessary information, difficulties in the digital payments, and weak doctor-patient relationships are also growing at a similar rate [28–31]. All of these problems contribute to the Pakistani’s healthcare system inefficiencies and ineffectiveness, which directly impact patient satisfaction.

The influence of laboratory services on patient satisfaction has been measured by Manzoor, Wei [32], which revealed LS positively connected to the PS. A study used pharmacy services to access the PS [33], also studies evaluated PS through physical facilities [34], and DPC [35]. Previous scholars examined PS with single or two variables, but this study used multiple factors such as LS, PHS, PF, DS, NS, DPC, RS, and digital payments with the transitions digital system for examining the patient study. As per the author's understanding, this framework is unique in the literature on healthcare services regarding the PS. Our study aims to investigate improved factors that contribute to patient satisfaction in Pakistani healthcare services, considering the development of the digital healthcare system. The research aims to give healthcare providers and policymakers an understanding of the unique determinants of patient satisfaction in Pakistan which will help them to identify the customer factors and develop their strategies for the opportunities to improve the quality of healthcare delivery in the country. Satisfaction of patients is one of the most important measures of healthcare quality and the potential ramifications on healthcare policy as well as practice are widespread. However, the existing literature offers minimal direction on the most effective ways to predict and evaluate patient satisfaction, and even fewer insights on the suitable methodologies to employ [36].

**Fig. 1:**
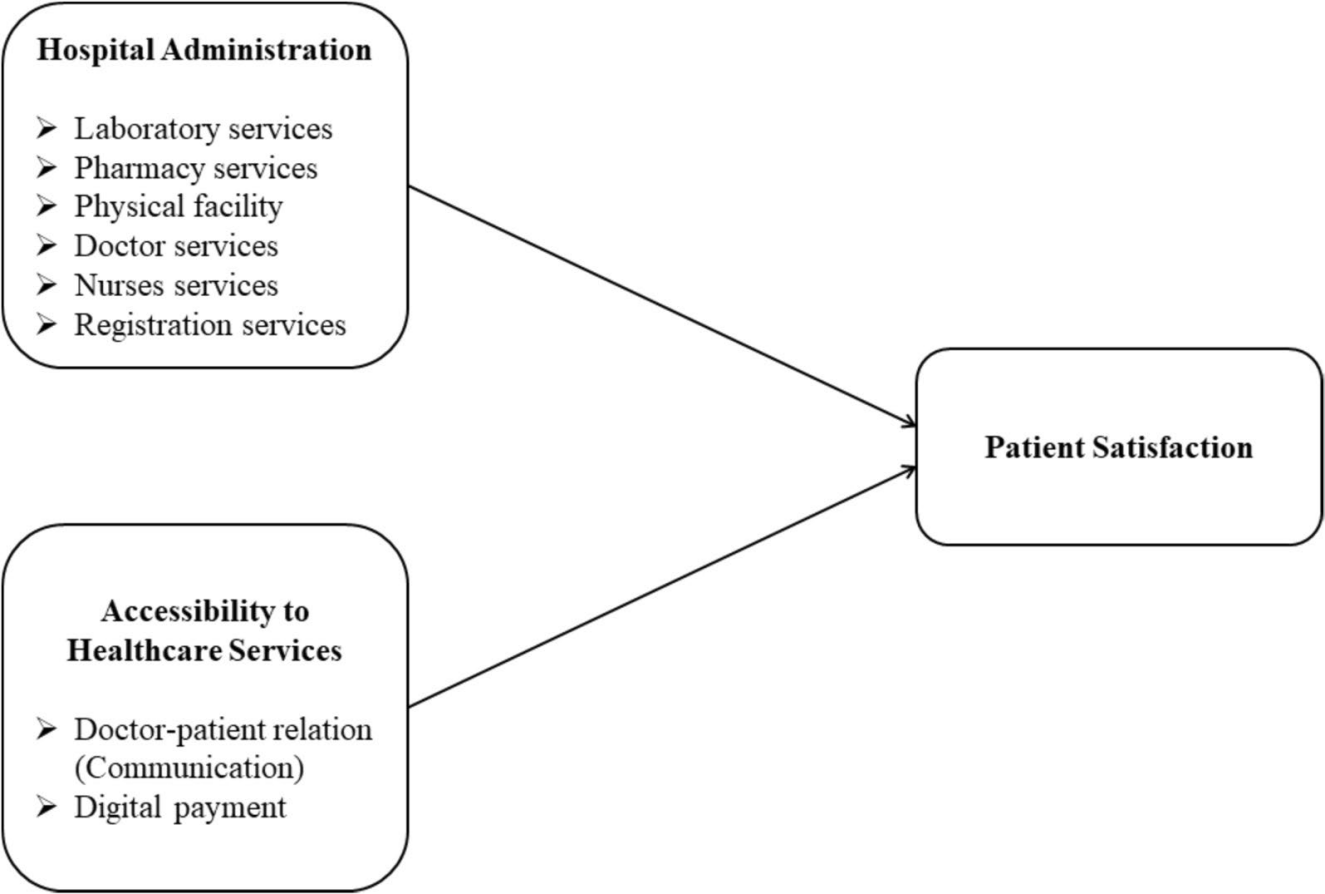
Conceptual framework

In Pakistan, the problem has been weakened by a lack of thorough research investigating the precise elements that affect patient satisfaction in healthcare institutions. It is essential to address these gaps to establish policies that would improve healthcare delivery and patient satisfaction in the country. The shortcomings identified above give rise to the following research aims. These domains include assessment systems, measure service provider performance, information sharing, and monitoring methods, which are necessary for the involvement and investment of both the public and private sectors. Establishing national and regional digital services is necessary for developing satisfaction among patients as well as facilitating the efficient and effective allocation of resources. By addressing these research gaps, this study can provide a more comprehensive understanding of the influence of hospital services on patient satisfaction during the transition to digital systems. Figure 1, illustrates the conceptual model of the study.

*Research questions*

1. How and to what extent does the health care administration adversely affect the supply the quality of service delivery in public hospitals in Pakistan?
2. What are the services and to what extent they are provided in hospitals to the patients during outpatient department (OPDs) timing in Southern Punjab?
3. Which key factors influence patients’ satisfaction during the transition to digital systems in public hospitals in Southern Punjab?

*Research objectives*

The basic objective of this research is to assess the level of patient satisfaction with the outpatient department (OPDs) services in district hospitals situated in the Bahawalpur division of Southern Punjab, Pakistan. While specific objectives are:

1. To evaluate the level of patient satisfaction with outpatient department (OPDs) services in terms of laboratory services, pharmacy services, physical facilities, registration services, doctor services, nursing services, doctor-patient relations (communication), and digital payment systems.
2. To describe the healthcare facilities in the OPDs and accessibility to services regarding online appointments, payments, and information about the health of patients who attended the OPDs in district hospitals.
3. To find the association between services provided by hospital administration connecting with the digital payment system with services, accessibility, and patient satisfaction concerning the OPDs facilities and propose a better measurement model for PS, according to the shift of digital system.

These research questions and objectives provide a structured approach to understanding and improving various aspects of the Pakistani healthcare system from the perspective of patient satisfaction.

### Hypothesis

**H1:** The more doctor services the patients receive, the more satisfaction they feel.

**H2**: The more physical facilities the patients receive, the more satisfaction they feel.

**H3:** The more nurse services the patients receive, the more satisfaction they feel.

**H4:** The more pharmacy services the patients receive, the more satisfaction they feel.

**H5:** The better the DPC the patients perceive, the more satisfaction they feel.

**H6:** The more easily registration the patients receive, the more satisfaction they feel.

**H7:** The more laboratory services the patients receive, the more satisfaction they feel.

**H8:** The more effective the digital payment system the patients perceive, the more satisfaction they feel.

### The Health Care System of Pakistan

**Fig. 2:**
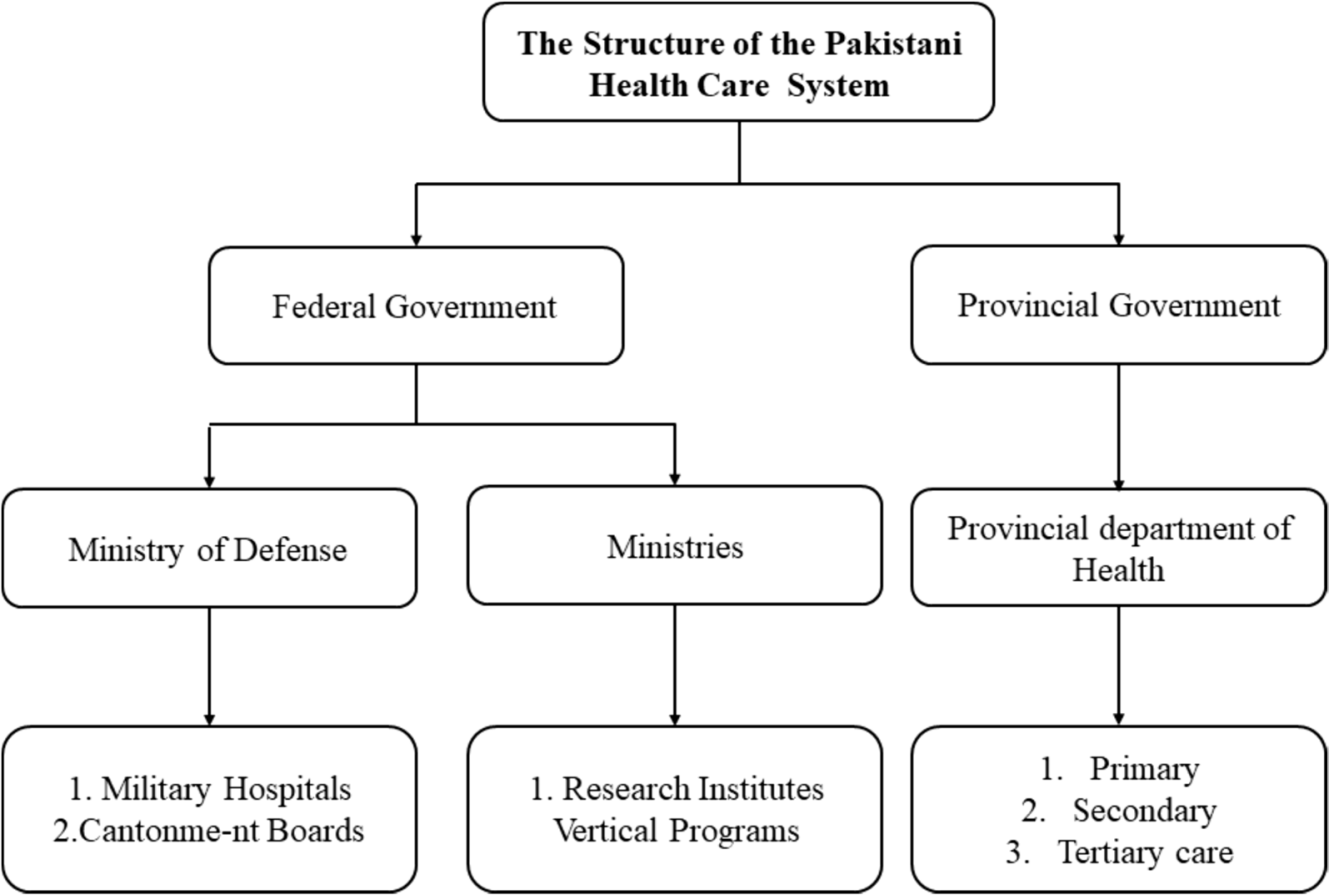
The public health care delivery system in Pakistan

**Fig. 3:**
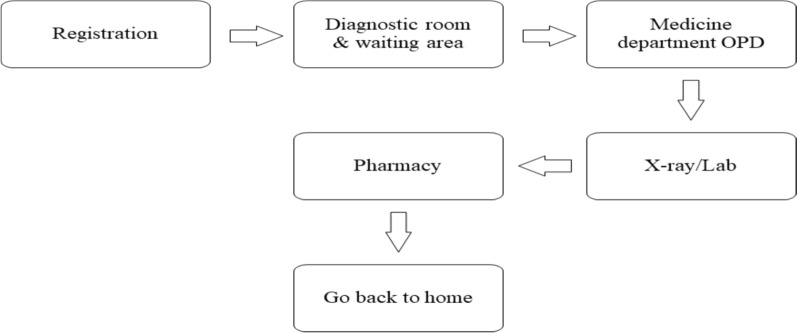
Process for accessing OPDs

Many different groups have participated in Pakistan's healthcare system, from public and private corporations to philanthropic foundations and foreign aid agencies. Promotion, prevention, treatment, and rehabilitation are the four components of Pakistan's healthcare system [30]. The constitution of Pakistan states that all residents have the right to obtain basic healthcare. The 18th amendment of the Pakistani constitution transferred the authority of healthcare policy development from the federal level to the provincial level. Each province is now accountable for formulating healthcare policies for the approximately 200 million citizens of the country. Figure 2 presents a comprehensive summary of the healthcare system in Pakistan that is accessible to the general public. There exist distinct public healthcare systems administered by both the federal and state governments [37], while Fig. 3, describes the process of accessing OPDs.

## Materials and methods

### Sample

The degree of patient satisfaction with laboratory facilities, pharmacy services, physical facilities, doctor services, nurse’s services, registration services, doctor-patient relations (Communication), and digital payment services was investigated through a quantitative survey. The research was carried out between March to May of 2023 in the public hospitals' outpatient departments in the Multan, Lodhran, Dera Gazi Khan, Bhawalnagar, Rahimyar Khan, and Bahawalpur districts of Southern Punjab, Pakistan. The questionnaire had two sections, the first section consisted of the demographic details, and the second one consisted of the standard of care provided by local hospitals concerning laboratory services, pharmacy services, physical facility, doctor services, nurse’s services, registration services, doctor-patient relation (Communication) and digital payment. By the recommendations provided by Krejcie and Morgan [38] and Saunders, Lewis [39], distributed 1000 questionnaires in the hospitals to get the responses. We obtained 879 completed responses for analyses with an 87% response rate. The medical staff and less than 20 years patients were excluded from the study (Tables 1, 2).

**Table 1:** Population details

Sr. no.	Population	Area km^2^
Punjab Province	110,012,442	205,344
Bahawalpur District	3,668,106	24,830
Bahawalnagar District	2,981,919	8878
Rahim Yar Khan District	4,814,006	11,880
Multan District	1,871,843	17,935
Lodhran District	1.7 Million	1790
Dera Ghazi Khan District	2,872,201	11,290

Source: Author compilation

**Table 2:** Hospital’s details of the study area

Sr. No.	Teaching hospital	District hospital (DHQ)	Tehsil Headquarter (THQs)	Rural Health Centers (RHCs)	Basic Health Units (BHUs)
1. BWP	1	0	4	10	72
2. BWN	0	1	4	10	101
3. RYK	1	0	3	19	104
4. MTN	1	1	4	08	82
5. LDR	0	1	2	04	48
6. DGK	1	0	2	09	51

Source: Author compilation

### Instruments

As per Saunders, Lewis [39], and Krejcie and Morgan [38] the sample size selected and their approaches were widely used in the services studies with reliable results. The research respondents consisted of six public hospital patients from outpatient departments in Bahawalpur, Bahawalnagar, Rahimyar Khan, Multan, Lodhran, and Dera Gazi Khan, South Punjab, Pakistan. This research adapted all constructs from the previous studies (see Appendix 1) and collected data through a random sampling technique. The 9 items were used to evaluate patient satisfaction (PS), from the study of Tucker and Adams [40]. The doctor’s services were accessed through 7 items and the nurse’s services were measured by 8 items from Xie and Or [41]. The digital payment system was evaluated through 3 items adapted from the Singh and Mudang [42], whereas registration services are measured with 4 items adapted from Khan, Aslam [43]. The pharmacy services were examined through 8 items adopted by Xie and Or [41], while laboratory services are accessed with 6 items adapted by Xie and Or [41]. Doctor-patient communication and physical facilities were measured respectively 4 and 5 items adapted from the study of Andaleeb [14]. The research utilized a 5-point Likert scale for assessing all constructs (excluding participants' information), where 5 = strongly agree and 1 = strongly disagree. The level of education, Age, and marital status were some of the demographic characteristics of the study population (see Table 3 for further details). The patients, who had little formal education, answered questions in their native language Sariki [44].

**Table 3:** Establishing the reliability of the study

Variables	CA coefficient
PS	1.439
PHY	1.522
DPC	1.413
LS	1.292
DS	1.633
NS	1.472
RS	1.317
DPS	1.742

### Establishing the reliability of the questionnaire

The current research applies a pilot study to determine the questionnaire's reliability. The pilot research consists of a sample size of around 200 people from the target population. A sample of responders consisted of 200 individuals, which represented approximately 22% of the total population. The Cronbach's alpha (CA) test is employed for the analysis of the findings from the pilot study. The study employed the CA approach with SPSS to assess the internal reliability. According to [45], a CA coefficient of 0.70 is considered good. The 0.7 criteria were developed based on observed patterns in social science research. Table 3 displays the constructions and the associated CA coefficient, which shows respondents understand the questionnaires.

### Analysis methods

The Social Science Statistical Package (SPSS), analysis of moment structures (AMOS), and structural equation modeling were used to analyze the data for reliability, validity, correlations, and descriptive findings. We performed confirmatory factor analysis (CFA) and multiple regression analysis tests to examine our hypothesized model. The use of multiple regression has been utilized to observe changes in the dependent variable (patient satisfaction) which is predicted by autonomous variables such as DS, DPS, NS, LS, PHS, RS, PF (environmentally and tangible), and DPC. The consistency analysis evaluated all items' reliability and authenticity [46]. Additionally, we created a measuring model that considered the relationships between the investigated factors and their items. We employed a three-stage approach to implement Qing's [26] suggested SEM approach. Firstly, the research employed a measurement model to evaluate component scores for all items. Secondly, discriminant validity was determined using CFA, and thirdly, the casual model was accessed through the regression SEM technique [47]. These measures were taken to ensure the causal model's validity and reliability.

The demographic details of 879 respondents can be seen in Table 4 and 413 were male, while 466 were female. The 119 participants (13.53%) belong to the age of 20–29, 153 participants (17.40%) belong to the age of 30–39, 296 patients (33.68%) belong to the age of 40–49 and 311 patients (35.39%) above the age of 50 years. The 107 participants (12.17%) had no formal education, the 223 patients (25.37%) had school education, 361 patients (41.07%) completed education from the college and 188 respondents (21.39) graduated from the university.

**Table 4:** Demographic details of the respondents

Description	No	Percentage
Gender		
Male	413	46.99
Female	466	53.01
Marital status		
Married	501	56.99
Unmarried	378	43.01
Age		
20–29	119	13.53
30–39	153	17.40
40–49	296	33.68
≥ 50	311	35.39
Education		
No formal education	107	12.17
School	223	25.37
College	361	41.07
University	188	21.39

## Results

### Common method bias (CMB)

The first use of the Harman single-factor approach and CMB was tested. This study shows that the 54-item measurement model had eight apparent factors. However, the single factor variance of the highest account was 32.07%, as indicated by Podsakoff, MacKenzie [48]. This is below the critical threshold of 40%; therefore, no CMB occurred. The variance inflation factors and VIFs were Kock [49], and the non-existence of CMB is confirmed as all VIFs are < 3.3. Further, to minimize probable distortion resulting from social desirability bias, steps first involved the identification of the respondents about the study and assurance of anonymity and confidentiality.

### Validity and reliability

The CFA validated the association between the observables and their contained latent variables. Items' loadings on a certain factor could be evaluated with its help. The results of the confirmatory factor analysis, including the factor loadings, are presented in Table 5. Appropriateness of fit criteria was met by the statistical values of the confirmatory factor analysis. The factor loadings' values indicated the significance of the factors' associations with the targeted constructs. This model's factor loading values ranged from 0.926 to 0.562. When the loadings of the items and their reliability were examined, all of the factor loading values supported the validation of the construct validity. The value of Cronbach’s alpha and composite reliability values are higher than 0.7 for the evaluation criteria (see Table 4), which suggests the internal consistency of the items within a construct [50].

**Table 5:** Results of the confirmatory factor analysis

Construct/factors	Items	Factor loadings	Cronbach’s Alpha
Patient satisfaction			**0.966**
	PS1	0.787	
	PS2	0.793	
	PS3	0.864	
	PS4	0.879	
	PS5	0.850	
	PS6	0.724	
	PS7	0.871	
	PS8	0.863	
	PS9	0.850	
Pharmacy services			**0.931**
	PHS1	0.799	
	PHS2	0.858	
	PHS3	0.824	
	PHS4	0.816	
	PHS5	0.802	
	PHS6	0.845	
	PHS7	0.809	
	PHS8	0.805	
Doctor–patient communication			**0.942**
	DPC1	0.647	
	DPC2	0.846	
	DPC3	0.837	
	DPC4	0.807	
Laboratory services			**0.927**
	LS1	0.808	
	LS2	0.790	
	LS3	0.815	
	LS4	0.782	
	LS5	0.699	
	LS6	0.562	
Physical facilities			**0.968**
	PF1	0.920	
	PF2	0.922	
	PF3	0.868	
	PF4	0.864	
	PF5	0.917	
Nurses services			**0.925**
	NS1	0.752	
	NS2	0.801	
	NS3	0.782	
	NS4	0.734	
	NS5	0.787	
	NS6	0.819	
	NS7	0.799	
	NS8	0.736	
Doctor services			**0.820**
	DS1	0.713	
	DS2	0.815	
	DS3	0.785	
	DS4	0.818	
	DS5	0.803	
	DS6	0.792	
	DS7	0.841	
Registration services			**0.895**
	RS1	0.926	
	RS2	0.861	
	RS3	0.860	
	RS4	0.010	
Digital payment system			**0.737**
	DP1	0.795	
	DP2	0.803	
	DP3	0.745	

Table 6 describes the analyses of each factor’s convergent validity, discriminant validity, and composite reliability were also measured to ensure the data and the model were consistent with one another. The value of composite reliability (CR) > 0.7, average variance extracted (AVE) > 0.5, and CR > AVE, which suggests the convergent validity of the constructs [51].

**Table 6:** Composite reliability, convergent, and discriminant validity

V.N	C.R	AVE	MSV	PS	PHS	NS	DS	PF	LS	RS	DPC	DPS
PS	0.963	0.742	0.551	**0.861**								
PHS	0.921	0.596	0.472	0.038†	**0.772**							
NS	0.921	0.595	0.393	0.559***	− 0.059†	**0.771**						
DS	0.938	0.686	0.511	0.495***	0.032†	0.391***	**0.828**					
PF	0.967	0.855	0.587	0.293***	0.028†	0.218 ***	0.429***	**0.925**				
LS	0.883	0.578	0.338	0.431***	0.011†	0.516***	0.424***	0.224***	**0.760**			
RS	0.940	0.799	0.597	0.229***	0.093**	0.193***	0.203***	0.295***	0.188***	**0.894**		
DPC	0.867	0.624	0.428	0.016†	0.556***	− 0.056†	0.017†	0.019†	0.045†	0.035†	**0.790**	
DPS	0.777	0.537	0.353	0.291***	0.030†	0.155***	0.309***	0.325***	0.198***	0.388***	0.082***	**0.733**

*PS* patient satisfaction, *PHS* pharmacy services, *LS* laboratory services, *PF* physical facilities, *DPC* doctor–patient communication, *DS* doctor services, *NS* nurses services, *REG* registration services, *DP* digital payment system. *CR*  composite reliabilities, *AVE*  average variance extracted, *MSV*  maximum shared variance

Significance level: ***p < 0.001

Furthermore, to measure the discriminant validity of the constructs, the extent to which various constructs deviate or diverge from one another, we used two criteria; first, AVE > maximum shared variance (MSV), and second, the square root of AVE for each construct (diagonal values of the correlation matrix in Table 6) should be greater than the absolute value of inter-construct correlations (off-diagonal elements) [52, 53]. Thus, the AVE > MSV and the square root of AVE values at a diagonal in Table 5 is greater than any correlation between the constructs, hence suggesting the discriminant validity of the constructs—these results for the measurement model support the use of the proposed model in this study. The current research results exhibited all α reliability coefficients (PS = 0.95, PHS = 0.93, LS = 0.92, PF = 0.91, DPC = 0.97, DS = 0.92, NS = 0.96, RS = 0.93, and DP = 0.78), which are greater than the value of 0.70 [54]. The values of means, standard deviations (SD), correlations, and “α” reliabilities for each variable can be seen in Table 7.

**Table 7:** Descriptive statistics, Pearson’s correlations, and reliability coefficients

Variable	Mean	SD	1	2	3	4	5	6	7	8	9
PS	4.01	0.632	**(0.95)**	0.496^**^	0.483^**^	0.440^**^	0.290^**^	0.472^**^	0.468^**^	0.221^**^	0.264^**^
PHS	4.13	0.684		**(0.93)**	0.511^**^	0.373^**^	0.216^**^	0.824^**^	0.351^**^	0.230^**^	0.164^**^
LS	3.94	0.600			**(0.92)**	0.399^**^	0.257^**^	0.487^**^	0.438^**^	0.196^**^	0.214^**^
PF	3.52	0.907				**(0.91)**	0.350^**^	0.328^**^	0.858^**^	0.176^**^	0.223^**^
DPC	3.01	0.902					**(0.97)**	0.220^**^	0.450^**^	0.317^**^	0.291^**^
DS	3.87	0.736						**(0.92)**	0.326^**^	0.186^**^	0.171^**^
NS	3.54	0.768							**(0.96)**	0.199^**^	0.261^**^
RS	2.22	0.789								**(0.93)**	0.363^**^
DPS	2.85	0.821									**(0.78)**

*PS* patient satisfaction, *LS* laboratory services, *DPC* doctor–patient communication, *PF* physical facilities, *DS* doctor services, *NS* nurses services, *RS* registration services, *DP* digital payment system

**Correlation is significant at the 0.01 level (two-tailed)

#### Model fit

The values of the standardized root mean square residual (SRMR) = 0.049, the comparative fit index (CFI) = 0.944, the normed fit index (NFI) = 0.924, the Tucker-Lewis index (TLI) = 0.939, the relative fit index (RFI) = 0.917, the incremental fit index (IFI) = 0.945 and the root mean square error of approximation (RMSEA) = 0.054 as well as values are suggested standards by scholars [61, 64–66]. Model fit indices valves can be seen in Table 8 which meet the recommended standard by the [55].

**Table 8:** Model fit statistics

Absolute model fit indices	
Chi-square	4576.199
DF	1301
Chi square/DF	3.517
Standardized Root Mean Residual (SRMR)	0.049
Comparative Fit Index (CFI)	0.944
Normed Fit Index (NFI)	0.924
Tucker Lewis Index (TLI)	0.939
Relative Fit Index (RFI)	0.917
Incremental Fit Index (IFI)	0.945
Root Mean Square Error of Approximation (RMSEA)	0.054

### Hypothesis testing

The multiple regression analysis was conducted to understand factors that have effects on patient satisfaction in public hospitals, which result can be seen in Table 9. The findings indicated that the 8 independent variables (pharmacy services, laboratory services, doctor-patient communication, physical facilities, doctor services, nurses' services, registration services, and digital payment) accounted for 38.7% (Adjusted *R*^2^ = 0.387, *F* = 68.025, and *p* = 0.000) of the variance in the outcome variable (patient satisfaction). According to the data in Table 8, five of the eight variables have a positive and significant effect on patient satisfaction, while the other variables fail to reach statistical significance. Patient satisfaction increases by 0.014 units for every unit that doctor services increase (*β* = 0.118; *t* = 2.455, and *p* < 0.00). Enhanced patient satisfaction is achieved by improving the quality of medical treatments provided by doctors. Specifically, enhancing the standard and availability of services through digital systems as well as appointments and advice about following the prescription is an essential aspect of building patient satisfaction. The nurses' services (*β* = 0.180; *t* = 3.217, and *p* < 0.00) show that for every unit increase in nurses' services, patient satisfaction rises by 0.256 units. PS improves considerably with quality nursing care. Results point to the importance of attention and professional nursing care for patient satisfaction. Nurse training, appropriate nurse-to-patient ratios, and supported work environments for nurses are all linked to improved patient satisfaction, so investments in these areas also make an appropriate decision for authorities.

**Table 9:** Multiple regression models (dependent variable: patient satisfaction)

Variables	β Coefficients			95.0% Confidence interval for β		Collinearity statistics	
	*Β*	*T*	Sig.	Lower bound	Upper bound	Tolerance	VIF
DS	0.118	2.455	0.014	0.020	0.182	0.313	3.194
PF	0.063	1.183	0.013	− 0.029	0.117	0.255	3.919
NS	0.180	3.217	0.237	0.058	0.239	0.230	4.355
PHS	0.185	3.732	0.001	0.081	0.260	0.295	3.391
DPC	0.037	1.170	0.000	− 0.018	0.069	0.723	1.382
RS	0.027	0.896	0.242	− 0.026	0.069	0.799	1.252
LS	0.193	5.777	0.371	0.134	0.272	0.648	1.544
DPS	0.090	3.032	0.000	0.025	0.115	0.812	1.232

*PS* patient satisfaction, *PHS* pharmacy services, *LS* laboratory services, *DPC* doctor–patient communication, *PF* physical facilities, *DS* doctor services, *NS* nurses services, *RS* registration services, *DP* digital payment

Additionally, patient satisfaction rose by 0.001 units for every unit of pharmacy services performed (*β* = 0.185; *t* = 3.732, and *p* < 0.00). The quality of pharmacy service directly impacts patient satisfaction. Enhancing the quality of these services, even to a small extent, will contribute to delivering a superior experience for individuals receiving patient care. It is recommended to relocate the pharmacy to the Hospital and ensure the accessibility of medications, regardless of the prescription, while minimizing patient waiting time. Patient satisfaction increased by 0.371 units for every unit that laboratory services increased (*β* = 0.193, *t* = 5.777, and *p* < 0.00). The level of patient satisfaction is closely tied to the quality of laboratory services provided. The significance of positive laboratory results in enhancing patient care should not be ignored. The problem of ensuring the reliability of laboratory services is resolved by acquiring the fundamental set of laboratory equipment and implementing methods to expedite the delivery of samples to and from the laboratory. An increase of one unit in digital payment is shown to boost patient satisfaction by 0.091 units (*β* = 0.090; *t* = 3.032, and *p* < 0.00). Improving patient satisfaction by providing digital payment options, indicates that consumers value the ease of digital transactions. Hospitals using or encouraging digital pharmacy payment systems to simplify transactions, and estimates showing billing errors could be lowered and wait times shortened for patients if more widescale adoption of electronic payments were achieved. Physical facilities (*β* = 0.063, *t* = 1.183, and *p* > 0.014) found an insignificant impact on PS, although the study indicates that the PF is important to improving satisfaction, it might not be the only reason. Even though it is a relatively small factor in this case, clean, safe, and comfortable facilities still matter for the patient experience as a whole and cannot be ignored.

Doctor-patient communication (*β* = 0.037, *t* = 1.170, and *p* > 0.05) was also found insignificant, DPC leads to no change in patient satisfaction according to a study, one answer is that it reflects differences in the other aspects of variability in ways of communicating. The DPC might still be an important aspect for hospitals to focus on and improve and may improve other facets of care as well. This might indicate the usefulness of training programs on effective communication techniques for doctors. Patient satisfaction was not highly related to registration services (*β* = 0.027, *t* = 0.896, and *p* > 0.242). This would indicate that the initial administrative step may not be quite as important as the medical services rendered. Finally, it may seem like a small thing but maintaining proper patient flow and a quick registration process through the digital way will help avoid frustration at the outset of a patient's visit and can help enhance the patient experience overall. The study's findings indicate that H1, H3, H4, H7, and H8 were confirmed, but H2, H5, and H6 were not supported.

## Discussion

The study results significantly connected with the services of public hospitals which influenced patient satisfaction as well as the unfolding performance of the healthcare institutions. Provision of medical care including doctors and nurses, quickness in pharmacy, efficient laboratory operations, and digital payment options greatly enhance patient satisfaction to a large extent. In our research, we found the levels of patient satisfaction in various dimensions of doctor services. The findings reveal that some essential dimensions tend to affect the way patients feel and evaluate their experiences. Patients consistently expressed increased satisfaction when provided with proper diagnosis and beneficial treatments. This is consistent with other studies, which suggest that the essential element of patient trust and satisfaction is clinical competence [56]. Patients also found more satisfaction when their doctors appeared respectful, confidential, and ethically appropriate. The high standards of professionalism in a doctor are the building blocks to a successful relationship between the patient and doctor [57]. The study found a significant association between the quality of care delivered by doctors and patient satisfaction with healthcare professionals in Pakistan. Similarly, studies from Malawi [58] Nigeria [59] Saudi Arabia [60], illustrated that doctors' services are an influential factor for patient satisfaction because service providers' abilities directly affect satisfaction. According to the research of Iran [61], doctor services are the most important aspect in determining outpatient satisfaction with service delivery. Our study supports the multidimensionality of the services provided by a doctor and their consequent impact on patient satisfaction. Improvement in these key dimensions can thus enhance overall patient experience and boost satisfaction levels.

The relationship between nursing services and patient satisfaction is an essential area in health care delivery in outpatient departments (OPD) [6]. Patient satisfaction is highly correlated with the quality and efficiency of nursing care since many patients visit the OPD, requiring quicker and more effective interaction. High-quality care includes thorough assessment, accurate documentation, and effective treatment plans [62]. Effective communication forms the backbone of the OPD clinic, as it is a place where encounters are short but highly crucial. Patients must be informed, in unambiguous terms and sensitively, of their health status and treatment plans. Patients come to an outpatient clinic for convenience so any delay will be a disappointment for them. OPD settings expect emotional support and empathy from HCPs because most patients are anxious and present with some apprehension regarding their health conditions [63].

Thus, nurses at the OPD should educate them about the health conditions and all possible treatment options. Proper patient education ensures better health outcomes and satisfaction. Personalized care, as per the needs and preferences of the patient, is another necessity in the OPD settings; it helps the patient feel valued and understood [64]. Nursing services form the basis for satisfying patients in OPD settings. Quality of care, communication efficiency, timeliness, emotional support, personalized care, and patient education are some of the critical factors [65]. These are the factors that, if improved in the various OPD healthcare facilities, will elicit a very high level of patient satisfaction from these patients. Indeed, these references have elaborated on the wide-ranging effect nursing services have on patient satisfaction in OPD settings and have pressed for continuous improvement in these areas to meet patient expectations and enhance healthcare quality overall [66].

This further implies that there is a relationship between the perceived effectiveness of digital systems of payment and satisfaction levels by patients. It would mean that if these digital payment methods are found to be very effective, efficient, user-friendly, and reliable by patients in many health facilities, then in general, the satisfaction level with the service increases. Additionally, a study revealed that in the US, many hospitals have adopted digital patient payment methods. It provides them with the opportunity to pay medical bills using an e-wallet or bank card both on the website and mobile application of a specific hospital. This study also shows that 74% of the patients reported higher satisfaction after applying digital payments [67]. Similarly. DP has cut down the time required to bill significantly and has increased the patient satisfaction level, with its start in most hospitals in India. For instance, the hospital has put in place a comprehensive digital payment system, following which its patient satisfaction scores have grown by 30% [68]. In addition, Safe, digital payment systems in hospitals in Germany [69], Kenya [70], Brazil [71], and Europe [72] assure protection against billing errors with a reduced chance of fraud. The advancement of mobile payment platforms, such as Alipay and WeChat Pay in Chinese hospitals substantially increased patient accessibility [73]. All studies support H8, which shows that digital payment systems in OPD positively affect patient satisfaction levels in many countries. The convenience, transparency, accessibility, and security maintained by such systems enhance the overall patient experience, which increases satisfaction, contributing to higher healthcare outcomes.

Pharmacy services affect patient satisfaction at a higher level through multi-dimensional healthcare delivery. These are areas critical to the contribution of PHS to increased patient experience and patient satisfaction in a more elaborate and evidence-based manner. The findings align with those conducted in India, which demonstrated that pharmacy services have a substantial impact on patient satisfaction [74]. A study Aziz, Fang [75] revealed that pharmacists ensure the proper medications are dispensed, identify potential drug interactions, and offer total medication reviews. These practices greatly eliminate medication errors and enhance patient safety, raising satisfaction levels. This includes counseling on how to use the medications, their potential side effects, and how to maintain the doses. Personalized counseling gives the patient a feeling of empowerment, which leads to overall satisfaction [76].

Further, the satisfaction of patients comes with the availability of pharmacies, accessibility of the pharmacist for consultation, and location of the pharmacies. Pharmacists contribute to public health through activities that involve health promotion and prevention of diseases, such as conducting campaigns for vaccination, smoking cessation programs, and wellness counseling [74]. The work not only benefits public health by yielding better outcomes, but it also improves patients' satisfaction by receiving comprehensive care beyond the traditional purview of medication management. Integrating pharmacists into healthcare teams automatically ensures a holistic approach to patient care. By collaborating with physicians, nurses, and other healthcare providers, pharmacists assist in designing comprehensive care plans and hence contribute toward improved patient outcomes and patient satisfaction [77].

Technology has been integrated into several medical activities, including laboratory services, research, and the design of new diagnostic equipment [78]. Laboratory services have played a major part in combating COVID-19, serving as the essential foundation for controlling the outbreak. Without these services, effectively managing the spread of the virus would have been unattainable [79]. Due to a huge population and understaffed departments, laboratory services at public hospitals in Pakistan are severely stressed, resulting in long waiting times for patients to receive their diagnoses [80]. This study found that the provision of laboratory services has a positive effect on patient satisfaction. This finding is consistent with previous research conducted in India [81] and Ethiopia [82]. The current study addresses the knowledge gap by assessing patient satisfaction concerning physical services, registration services, and doctor-patient communication. Patient satisfaction in Pakistan was not significantly affected by physical services, registration services, or doctor-patient communication. According to earlier research from Iran [83], South Korea [84], and India [85], where physical facilities had no discernible relationship with patient happiness, the facilities of physical in the outpatient department (OPD) had a very modest effect on satisfaction.

Patients' trust in the public health system as a whole will increase as a result of the implementation of corrective measures in these aspects. As per the findings, the majority of patients in Pakistan have a negative experience with doctors, nurses, and wait times, and a moderate experience with doctor-patient communication, physical services, and registration services. The findings indicate that incorrect policies and poor execution of doctor-patient communication, physical services, and registration services, ultimately lead to decreased patient satisfaction. Continual professional development programs for registration personnel are mandatory. Authorities should prioritize and actively participate in enhancing registration services. In the absence of regular maintenance, the availability of potable water is compromised, and the condition of the restrooms is extremely poor. Hence, the administration must prioritize these concerns and promptly resolve them by improving the infrastructure. Access to clean water and a hygienic environment is essential for the majority of the population. Implementing the proper care and maintenance protocols would enable healthcare facilities to enhance services and enhance patient satisfaction. This study discovered that the doctor-patient relationship had minimal impact on the patient's assessment of the quality of care they were provided. The World Health Organization (WHO) prioritizes sustainable development goals (SDGs) and recognizes the importance of service providers.

It also highlights the necessity for healthcare administration to evaluate its policies concerning service providers.

Problems related to the doctor–patient relationship of patients in public sector hospitals in Pakistan. Patients usually have a lot to wish to talk about their health. People curious about their health makes it easy for doctors to understand people. Doctors should be compassionate and helpful toward the suffering patients who go to hospitals to seek treatment. The increase in doctor-patient contact increases patient satisfaction with medical facilities. The significant determinants of patient satisfaction with healthcare services at hospitals in Punjab, Pakistan, include the availability of doctors and nurses, pharmacies and laboratories, physical and digital payments, and doctor-patient contact (relationship). Staff performance in effectively carrying out duties and tasks is essential for service quality in public hospitals. Timely delivery of health care improvements and patient satisfaction services were referred to as the fundamental parameters of service quality by the hospital.

## Conclusions and recommendations

A nation's prosperity is believed to be closely linked to its state of well-being. Currently, digitalized hospitals play an essential function in the diagnosis, treatment, and management of various illnesses and disorders. The hospital's inefficiencies would have a significant negative impact on society. The present authorities are struggling to the provision of good healthcare to patients in Pakistan. To achieve this objective, teaching hospitals and health service universities at the federal, provincial, and district levels are prioritizing the provision of patient-centered care in the numerous institutions around the country. To ensure that medical workers should consistently focus on patient-centered care and remain vigilant, as well as the institution should implement a new regulation regarding prompt response and prominent display of the patient charter in public.

However, there appears to be a lack of dedication to this intervention. For healthcare providers to effectively provide patient-centered care in Pakistani hospitals, it is necessary to implement comprehensive health system interventions that address financing, planning, service delivery, monitoring, and evaluation. In the most populous province of Pakistan, the present ratio of doctors to nurses is 67372:0.06124, while the ratio of nurses to the entire population is 7135:0.0006486 [86].

Implement regular training sessions for medical and non-medical personnel to enhance their communication skills, focusing on empathy, active listening, and clear explanations of medical conditions and treatments. Establish a system where patients can provide feedback on their interactions with doctors. Use this feedback to identify areas for improvement and tailor training programs accordingly. Simplify the registration process through a digital system by reducing paperwork and automating as many steps as possible. Introduce patient navigators to assist patients with the registration process, providing guidance and support to enhance their overall experience. Organize regular training of medical and non-medical personnel in communication skills with particular emphasis on empathy, active listening, clear explanation of the medical condition, and treatment. Put in place a system to receive patient feedback on their experiences. Analyze the feedback to enable refining of areas of improvement and support training programs. Simplify the registration process using a digital system that will minimize paperwork and automate as many steps as possible. Patients could be given navigators who would help them through the registration process, guiding and supporting them to improve their experiences.

Invest in upgrading the physical infrastructure in a way that would relate to waiting bays, consulting rooms, and washrooms to clean, comfortable standards for a healing environment. Further, the facilities should welcome all types of patients, including those with disabilities. This can involve changes such as ramps or even an elevator for access purposes and clear signage with directions to the restroom. Maintain the hospital surrounding regularly Make sure that digital payment systems are completely connected with all other hospital management systems which will help in smooth payments with minimized mistakes and errors. Create intuitive digital payment interfaces that are accessible and easy to use for patients at any stage in life. Improve security measures, so patients know why and that their data and financial information are safe, which can help you build trust in the payment system digitally.

Efficient clinical laboratory operations drive shorter times to test results Continuously Evaluate and Improve the Usability of Digital Systems to Meet the Needs of Staff and Patients Educate the healthcare staff on the use of digital systems to minimize errors & become more efficient by delivering rigorous training. Assist in support services to traverse digital systems for patient use, and enable patients to access their electronic health records (EHR) or book appointments without hurdles online. Implement high-quality inventory management systems to manage stockouts of essential medicines and queue time wastage for patients in need of medical services. Give patients specific directions and counseling regarding their medications, including potential side effects, and the importance of taking their prescribed treatments. Implementation of these recommendations will lead to improved patient satisfaction in OPDs of the Pakistani health care system. Focus on better communication, streamlined registration & digital payment processes, physical infrastructure upgrades, and quality standards in doctors' services, nurses' assistance, lab tests and pharmacy services will result in overall guided healthcare delivery to a more patient-centric experience.

Thus, this research provides a valuable perspective to the knowledge base on healthcare delivery systems in Pakistan and evaluates patient satisfaction in the health services context. This study makes a significant contribution to the literature because there are rare, published studies on patient satisfaction connected to digital service with healthcare facilities in Pakistan. It offers locally grounded insights that are essential to understanding the specific context, challenges, and opportunities for health services delivery in Pakistan. Briefly, this research supplements the existing health literature base by providing an extensive more-dimensional analysis of patient satisfaction with health services in Pakistan. Furthermore, this study raises important questions for future policy and research on a basis that measures quality and can be used to monitor trends in health in Pakistan.

### Theoretical implications

The patient satisfaction evaluation in health service delivery systems of Pakistan offers a rich context to explore theoretical implications across different domains, including service quality, health care management, and shifting services toward technology. The study investigates the influence of healthcare system components DS, DP, NS, LS, PHS, RS, PF, and DPC, on patient satisfaction. As the results indicate, LS, PHS, DS, NS, and DP impact patient satisfaction significantly, and therefore, those are the components that could be enhanced and focused on, while DPC, RS, and PF do not have a significant influence. The strong positive impact of digital payment systems (DP) means that efficiency is realized in the process of delivering healthcare. This suggests that improved service performance and patient satisfaction arise from integrating technology into the processes of offering health services. The close influence of doctor and nurse services (DS and NS) shows that the health system should be patient-centered to influence satisfaction. Patient-centeredness calls for healthcare providers to provide compassionate and effective care. The insignificant outcome of doctor-patient communication (DPC) on patient satisfaction shows a problem with the communication process, and the Health Communication Theory can be used to analyze these problems. The results simply imply that there has been a huge communication gap between doctors and patients, which indicates that the application of effective communication strategies has not been very proper.

This underlines the necessity to train healthcare providers in effective communication techniques to improve patient satisfaction. This suggests that investment in the physical infrastructure and environment in a healthcare setting will improve patient satisfaction. Investment shall be made to ensure that all the patients' expectations are met by putting modern facilities and equipment in place. Effective allocation of resources and process optimization is important in improving the registration services for a smooth flow of patients and reduced waiting time. The theoretical implications of the study explain patient satisfaction in the healthcare system of Pakistan by reviewing theories on service quality, health performance, technology acceptance, health communication, and infrastructure management. We get an illustrative picture of the factors involved in patient satisfaction. These results will help contribute not only to the academic discourse but also to providing practical guidelines for policy-makers and health administrators in general on the need to enhance healthcare quality to augment patient satisfaction in Pakistan.

### Practical implications

Essential health is the basic need of every human. Our proposed research has the potential to help the governments, concerned authorities, and hospital administrations to get rid of the increasing problems in health services in Pakistan, especially in Southern Punjab. According to the growing population and patients' demands, doctors and professional nurses should be employed to facilitate patient satisfaction. The staff should be well-trained to interact carefully with the patients. Beyond this, the registration and administrative process shall also be made less time-consuming and more accessible for patients. The hospital administration should focus on features like courteous staff, query handling at the reception counter, cooperative behavior of registration staff, and an efficient system of handling complaints. Our research findings offer relevant information on health administration that provides sufficient doctors and nurses with friendly patient management. The administrative staff could be trained to make a pleasant environment by recruiting a more significant number of doctors and nurses, which would mean less time in service availability and could also result in greater final patient satisfaction. Key measures would be to improve the physical infrastructure of hospitals, especially for disabled, bedridden, and elderly patients. Provisions should be made to increase the number of information desks, sign boards, prominently written instructions, and color-coding systems for those patients who are visiting the hospital for the first time. The most critical step to be taken is that the patient-to-staff ratio must be enhanced according to the standards in developed countries. Lastly, incorporating different social services networks into hospital service provisions will significantly improve the quality of care provided and achieve higher scales of patient satisfaction.

## Limitations

Moreover, this study is subject to various limitations. This study focused on the Bahawalpur division, which consists of six district hospitals located in southern Punjab, Pakistan. Furthermore, the data were collected between the hours of 9:00 am and 2:00 pm, namely during the operational hours of the hospital's outpatient department (OPD). The third constraint is that the data exclusively pertain to patients who visit the outpatient department (OPD). The study's conclusions and consequences cannot be generalized to the entire healthcare or service industries. One further constraint is that the research only included a limited number of participants from public hospitals, as a result of the random sampling procedures employed. Future research could explore the factors that affect patients' satisfaction with hospital care by increasing the sample size and implementing different sampling methods. To gain a more thorough understanding of the aspects that impact patients' satisfaction levels about tertiary health issues, researchers can strive to analyze additional essential factors, such as a disease-specific evaluation.

### Supplementary Information

Supplementary Material 1.

## Data Availability

The raw data supporting the conclusions of this article will be made available by the authors, without undue reservation.

## Appendix

**Table:**

	*Laboratory services (LS)*
LS1	Received the test results in time
LS2	Procedures and tests explained well
LS3	I received an adequate explanation of any tests I had to undergo
LS4	The staff was caring
LS5	The staff responded immediately when called
LS6	Feels the comfort with the test
	*Pharmacy services (PHS)*
PHS1	The pharmacy staff seems to have a genuine interest in me as a person
PHS2	The pharmacist spends as much time as is necessary with me
PHS3	If I have a question about my prescription, the pharmacist is always available to help me
PHS4	The pharmacist is good at explaining things in a way that I understand
PHS5	I am confident that the pharmacist dispenses all prescriptions correctly
PHS6	The pharmacy services that I've received are just about perfect regarding digital payment
PHS7	The pharmacist usually explains the possible side effects that a new medication may cause
PHS8	I'm very satisfied with the pharmacy services that I receive
	*Physical facilities (PF)*
PF1	There was a pleasant atmosphere in the ward
PF2	Conveniently located washrooms
PF3	Toilet facilities were clean
PF4	Cabins/Wards were regularly cleaned
PF5	I had access to the apparatus and equipment that was necessary for my medical care
	*Doctor services (DS)*
DS1	The doctors showed commitment (care about me)
DS2	The doctors seemed to understand how I experienced my situation
DS3	The doctors were respectful of me
DS4	The doctors were competent
DS5	I have some doubts about the ability of the doctors who treat me
DS6	My doctors treat me in a very friendly and courteous manner
DS7	Doctors usually spend plenty of time with me
	*Nurses services (NS)*
NS1	The nursing staff were well-trained
NS2	The nursing staff treated you as an individual
NS3	The nurses and assistant nurses showed commitment (cared about me)
NS4	The nurses and assistant nurses seemed to understand how I experienced my situation
NS5	The nurses and assistant nurses were respectful toward me
NS6	The nursing staff were courteous
NS7	The nursing staff had a clean appearance
NS8	The nursing staff were disciplined
	*Registration services (RS)*
RS1	Services were provided efficiently
RS2	The staff was professional
RS3	Medical procedures were performed correctly the first time
RS4	Rules and regulations were strictly maintained
	*Doctor–patient communication (DPC)*
DPC1	Doctors are good about explaining the reason for medical tests
DPC2	The doctors were willing to answer any questions
DPC3	Sometimes doctors make me wonder if their diagnosis is correct
DPC4	Doctors sometimes ignore what I tell them
	*Digital payment system (DP)*
DPS1	DP is much more convenient and user-friendly as compared to the paper-based conventional payment system
DPS2	The digital payment system is quicker than the traditional payment system
DPS3	I trust the ability of digital payment systems to protect my privacy & Transactions
	*Patient satisfaction (PS)*
PS1	The services of the healthcare provided by OPD are convenient
PS2	I find it hard to get an appointment for medical care right away
PS3	I can get medical care whenever I need it
PS4	I have easy access to the medical specialist I need
PS5	I feel confident that I can get the medical care I need without being set back financially
PS6	I have to pay for more of my medical care than I can afford
PS7	The medical care, I have been receiving is just about perfect
PS8	I think my doctor’s office has everything needed to provide complete care
PS9	When I go for medical care, they are careful to check everything when treating and examining me

## Abbreviations

DS: Doctor services
DP: Digital payment
NS: Nurses’ services
LS: Laboratory services
PHs: Pharmacy services
RS: Registration services
PF: Physical facilities
DPC: Doctor–patient communication
PS: Patient satisfaction
WHO: World Health Organization
OPDs: Out Patient Department
