# Supplementary material for: The influence of hospital services on patient satisfaction in OPDs: evidence from the transition to a digital system in South Punjab, Pakistan
Source: Health Res Policy Syst. 2024 Aug 5;22:93. doi: 10.1186/s12961-024-01178-8 (PMC11302220; doi:10.1186/s12961-024-01178-8)
Supplement: Supplementary file 1 — Supplementary Material 1. [file 12961_2024_1178_MOESM1_ESM.docx]

Appendix.1

|  | **Laboratory Services (LS)** |
| --- | --- |
| LS1 | Received the test results in time |
| LS2 | Procedures and tests explained well |
| LS3 | I received an adequate explanation of any tests I had to undergo |
| LS4 | The staff was caring |
| LS5 | The staff responded immediately when called |
| LS6 | Feels the comfort with the test |
|  | **Pharmacy Services (PHS)** |
| PHS1 | The pharmacy staff seems to have a genuine interest in me as a person. |
| PHS2 | The pharmacist spends as much time as is necessary with me |
| PHS3 | If I have a question about my prescription, the pharmacist is always available to help me. |
| PHS4 | The pharmacist is good at explaining things in a way that I understand. |
| PHS5 | I am confident that the pharmacist dispenses all prescriptions correctly |
| PHS6 | The pharmacy services that I've received are just about perfect regarding digital payment |
| PHS7 | The pharmacist usually explains the possible side effects that a new medication may cause. |
| PHS8 | I'm very satisfied with the pharmacy services that I receive |
|  | **Physical Facilities (PF)** |
| PF1 | There was a pleasant atmosphere in the ward |
| PF2 | Conveniently located washrooms |
| PF3 | Toilet facilities were clean |
| PF4 | Cabins/Wards were regularly cleaned |
| PF5 | I had access to the apparatus and equipment that was necessary for my medical care |
|  | **Doctor Services (DS)** |
| DS1 | The doctors showed commitment (care about me) |
| DS2 | The doctors seemed to understand how I experienced my situation |
| DS3 | The doctors were respectful of me |
| DS4 | The doctors were competent |
| DS5 | I have some doubts about the ability of the doctors who treat me |
| DS6 | My doctors treat me in a very friendly and courteous manner |
| DS7 | Doctors usually spend plenty of time with me |
|  | **Nurses Services (NS)** |
| NS1 | The nursing staff were well-trained |
| NS2 | The nursing staff treated you as an individual |
| NS3 | The nurses and assistant nurses showed commitment (cared about me) |
| NS4 | The nurses and assistant nurses seemed to understand how I experienced my situation |
| NS5 | The nurses and assistant nurses were respectful toward me |
| NS6 | The nursing staff were courteous |
| NS7 | The nursing staff had a clean appearance |
| NS8 | The nursing staff were disciplined |
|  | **Registration Services (RS)** |
| RS1 | Services were provided efficiently |
| RS2 | The staff was professional |
| RS3 | Medical procedures were performed correctly the first time |
| RS4 | Rules and regulations were strictly maintained |
|  | **Doctor-patient Communication (DPC)** |
| DPC1 | Doctors are good about explaining the reason for medical tests. |
| DPC2 | The doctors were willing to answer any questions |
| DPC3 | Sometimes doctors make me wonder if their diagnosis is correct. |
| DPC4 | Doctors sometimes ignore what I tell them. |
|  | **Digital Payment System (DP)** |
| DPS1 | DP is much more convenient and user-friendly as compared to the paper-based conventional payment system |
| DPS2 | The digital payment system is quicker than the traditional payment system |
| DPS3 | I trust the ability of digital payment systems to protect my privacy & Transactions |
|  | **Patient Satisfaction (PS)** |
| PS1 | The services of the healthcare provided by OPD are convenient |
| PS2 | I find it hard to get an appointment for medical care right away |
| PS3 | I can get medical care whenever I need it |
| PS4 | I have easy access to the medical specialist I need |
| PS5 | I feel confident that I can get the medical care I need without being set back financially. |
| PS6 | I have to pay for more of my medical care than I can afford. |
| PS7 | The medical care, I have been receiving is just about perfect |
| PS8 | I think my doctor’s office has everything needed to provide complete care |
| PS9 | When I go for medical care, they are careful to check everything when treating and examining me |
